# Cycling-Based Telerehabilitation: Acceptability and Feasibility Study

**DOI:** 10.2196/71099

**Published:** 2025-09-10

**Authors:** Sara Arlati, Vera Colombo, Marta Mondellini, Roberta Nossa, Chiara Grasso, Mauro Rossini, Emilia Biffi, Alessia Fumagalli, Eleonora Diella, Eleonora Guanziroli, Nicole Sanna, Emilia Ambrosini, Marco Sacco, Franco Molteni

**Affiliations:** 1 Institute of Intelligent Industrial Technologies and Systems for Advanced Manufacturing, National Research Council of Italy, Via Previati 1/E, Lecco, 23900, Italy, +39 03412350202; 2 Department of Psychology, Università Cattolica del Sacro Cuore, Milano, Italy; 3 Scientific Institute, IRCCS Medea, Bosisio Parini, Italy; 4 Respiratory Unit, IRCCS INRCA (Italian National Research Centre On Aging), Casatenovo, Italy; 5 Valduce Hospital, Como, Italy; 6 Villa Beretta Rehabilitation Center, Costa Masnaga, Italy; 7 Department of Electronics Information and Bioengineering, Politecnico di Milano, Milano, Italy

**Keywords:** telemedicine, virtual reality, exergaming, aerobic exercise, usability

## Abstract

**Background:**

Telerehabilitation is a promising solution to provide continuity of care. Most existing telerehabilitation platforms focus on rehabilitating upper limbs, balance, and cognitive training, but exercises improving cardiovascular fitness are often neglected.

**Objective:**

The objective of this study is to evaluate the acceptability and feasibility of a telerehabilitation intervention combining cognitive and aerobic exercises.

**Methods:**

A virtual reality–based dual-task exercise exploiting a cycle ergometer was designed, developed, and integrated with a commercially available telerehabilitation platform. Patients with different conditions were enrolled and administered subjective questionnaires investigating attitudes toward technology, usability, technology acceptance, and subjective workload. Their therapists were interviewed, and adherence and performance data were analyzed.

**Results:**

In total, 26 patients with neurological or post-COVID symptoms were included. Their attitude toward technology (range: 0‐5) did not change after the training period (pre: 3.44 [IQR 0.63]; post: 3.50 [IQR 0.48]); the platform was rated usable and acceptable. Frustration and physical and mental workload were present, especially among younger participants. The adherence was moderate, but individual differences were present (0.59 [IQR 0.54]). The therapists highlighted the potential of remote rehabilitation programs but also identified some limitations.

**Conclusions:**

This study proved the feasibility and acceptability of a customized virtual reality–based telerehabilitation program allowing for the safe implementation of aerobic cycling-based dual-task training. The solution was judged meaningful for dehospitalized patients, although some environmental and technical barriers should be overcome to implement telerehabilitation more effectively.

## Introduction

Rehabilitation comprises a series of interventions aimed at optimizing the functioning of an individual; it can play an essential role in regaining or maintaining autonomy during activities of daily living and reduce the personal, social, and economic impact of several acute or chronic conditions. On the contrary, without regular rehabilitation care, individuals with neurological, rheumatological, musculoskeletal, or cognitive disorders may experience a worsening of their condition, reduced quality of life, and emotional distress [1].

In such a context, a possible solution for the sustainable implementation of continuity of care is telerehabilitation, i.e., the remote delivery of rehabilitation services using information and communication technologies [2-5]. Telerehabilitation increases accessibility to care services in remote areas and developing countries [6] and enables patients to engage in higher doses of therapy or repetition, thus improving their performance at impairment, activity, and participation levels [7,8]. Existing studies have proven that telerehabilitation can improve patients’ adherence and satisfaction [9], allowing clinical personnel to assist patients who cannot travel or have mobility issues and leading to improved clinical outcomes similar to those obtained with standard therapy [10].

The importance of such remote care-related services has been further emphasized during the COVID-19 pandemic, which hit Italy severely in February 2020 [11]. The emergency that arose significantly overloaded hospitals and led to halting or limiting several clinical services [12]. Such an interruption caused detrimental effects on many patients who were in need of rehabilitation, raising the need to develop efficient, safe, and long-term remote rehabilitative programs [13].

Currently, telerehabilitation can be administered via video and phone calls from a therapist or through a platform that provides the patients with videos, messages, emails, and links to educational materials. Also, it can be supported by virtual reality (VR), a digital technology that allows the patient to train in a computer-generated scenario [14]. VR could generally offer many advantages to rehabilitation interventions [15]: it allows for the simulation of ecological and controlled environments in which patients can train safely and promotes the transferring of the acquired abilities to real life [16,17]. Also, VR has been proven to enhance performance and contribute to keeping patients’ motivation at a high level [18,19]. Thus, it can play a key role in promoting adherence, especially for patients with chronic conditions and needing (life-)long treatments.

When COVID arose, several telerehabilitation services were commercially available on the market [2,4]. However, most of them provided only cognitive exercises; a few focused on upper and lower body muscle strength and mobility [20] but did not address lower limb coordination and cardiovascular fitness [21], which—instead—could be very helpful for patients with post-COVID syndrome and chronic conditions in general [22-24].

This work was conceived to respond to the lack of rehabilitation services during the second wave of the COVID pandemic (September to December 2020). In particular, it aimed to design and develop a novel VR-based exercise supporting lower body and cardiovascular training to complement the existing exercises dedicated to upper limbs and cognitive functions in a commercial telerehabilitation platform.

Afterward, the feasibility and acceptability of a customized telerehabilitation program, possibly including such an aerobic exercise, were assessed in different categories of patients.

The remainder of this paper is organized as follows. The Methods section describes the qualitative study performed; the Equipment subsection, besides technical aspects, presents the design choices and the rationale for developing the novel VR-supported exercise. The following sections contain, respectively, the study outcomes, their discussion, and the conclusions we drew with some recommendations for future work.

## Methods

### Study Design

The study presented in this work was a pilot, interventional, and multicentric study conducted between July 2022 and March 2023.

### Ethical Considerations

The study was approved by the Ethics Committees of Ospedale Valduce – Villa Beretta Rehabilitation Center (VB) (Prot. N. 150/2022; date of approval: February 24, 2022), IRCCS E. Medea (MEDEA) (Prot. N. 23/22 – CE; date of approval: March 31, 2022), IRCCS INRCA Casatenovo (INRCA) (Prot. N. 14/22; date of approval: May 26, 2022). All the procedures were performed under the Declaration of Helsinki. All adult participants gave their written informed consent. In the case of participants below legal age, informed written consent was obtained from their parents or legal guardians. No compensation for participation was given.

### Equipment

#### Requirements and Design

As this work was conceived to give an immediate response during the pandemic, the following three requirements were set: (1) the system must accommodate different conditions, including post-COVID patients, and include different exercises for motor, aerobic, and cognitive training; (2) it must be safe for home use; and (3) it had to be available in a short time.

The first two requirements were not easy to satisfy, as, in general, program interventions focusing on balance and gait were delivered in person due to the potential risks of falls [25]. Experiences reported in the literature either occurred while sitting or lying down or were dedicated to orthopedic patients with no other co-morbidities [20]. Furthermore, in most cases, the focus was on increasing muscle strength and mobility rather than promoting coordination and improving cardiovascular fitness [21].

We used a cycle ergometer to satisfy safety and controllability requirements and the need to support an aerobic training program for lower limbs. In this way, in fact, aerobic training could be carried out safely while seated and holding the cycle ergometer handlebars [26,27]. Moreover, using an ergometer allows for workload adjustment and, thus, the possibility of modifying the difficulty of the proposed task according to each patient’s characteristics [28]. Finally, cycling and walking share common features: both movements are periodical, require alternative joints’ flexion/extension, and require the contraction of agonist/antagonist muscles in a coordinated pattern [29]. To ensure safety related to the accomplishment of physical activity, additional physiological sensors were added to the system (i.e., heart rate (HR) and oxygen saturation) to interrupt the exercise when it was too exhausting.

To answer the third requirement, two elements were considered. First, we decided to use commercially available equipment already certified by Medical CE. Second, we investigated the availability of telerehabilitation companies to integrate a cycling-based task. These requirements were met by the cycle ergometer from COSMED (Rome, Italy) and the Virtual Reality Rehabilitation System (VRRS) produced by Khymeia Group (Padova, Italy). Both systems are further described in the following section, along with the developed virtual environment.

#### The Telerehabilitation System

The VRRS is a commercial telerehabilitation system composed of a tele-cockpit operated by the therapists in the clinics and a patient kit. The VRRS tele-cockpit allows for the creation of a daily rehabilitation plan and assessment of patients’ compliance and performance. Through the tele-cockpit, the therapist can also video call the patients to assist her or him during the exercise execution. The patient kit comprises a Windows Surface laptop (VRRS Home Tablet), wearable sensors, and interaction devices (e.g., inertial measurement units). The VRRS system offers, per se, several modules dedicated to rehabilitating specific functions, e.g., cognitive, speech, neuromotor, and respiratory exercises [30].

For the sake of this study, the VRSS system was integrated with medical-certified cycle ergometers and a custom-built VR environment. In total, 3 different models of cycle ergometers were used: E100, E5, and E150 Pediatric. All were equipped with a chest band with a HR monitor; E5 models also integrated an oximeter to measure oxygen saturation (SpO_2_).

**Figure 1.**
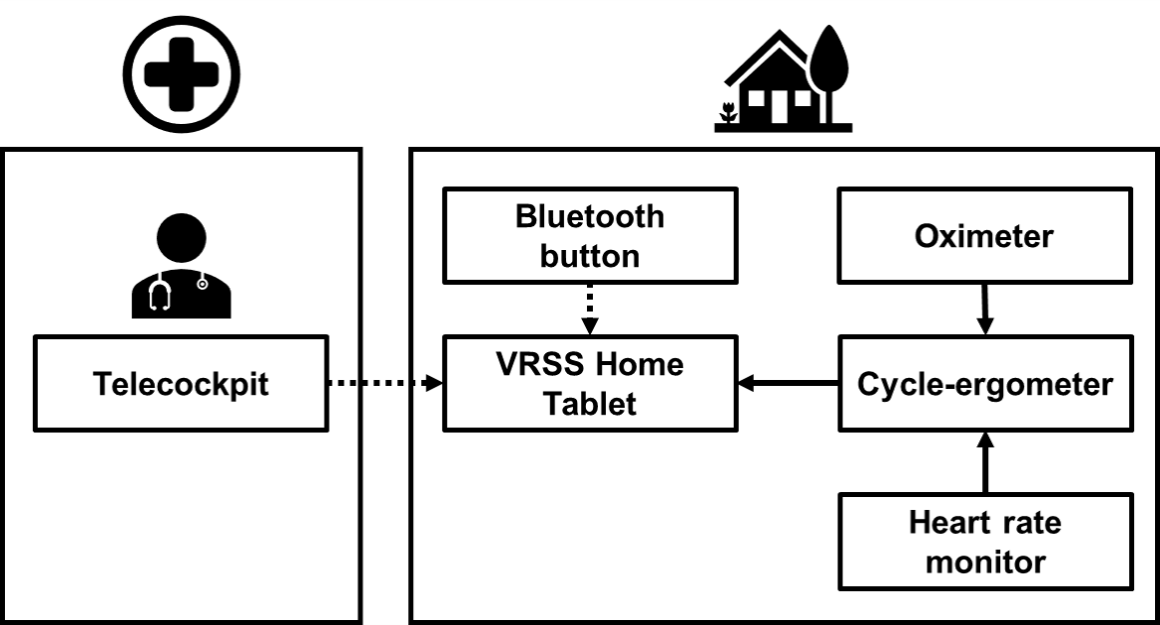
A schema of the ARTEDIA application as integrated into the Virtual Reality Rehabilitation System (VRRS) from Khymeia [26].

The VR environment designed and developed with Unity (Unity Technologies) to support dual-task training was named ARTEDIA and made available, among other VRSS applications, on the Home Tablet. The technical connection details shown in Figure 1 are further described in [26].

The exercise occurs in a virtual park where the patient can travel along a predefined path; the forward velocity is synchronized according to the current ergometer’s revolutions per minute (RPMs).

**Figure 2.**
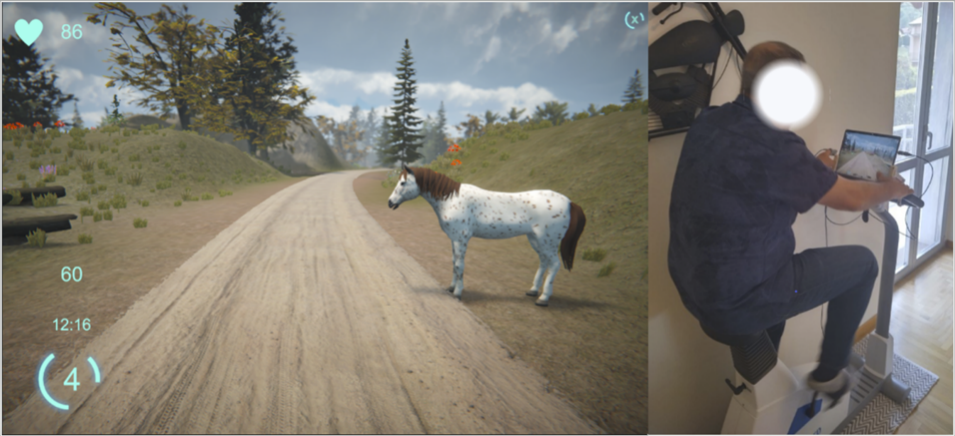
A screenshot of the ARTEDIA application with a no-go target appearing along the route. On the right side, a patient doing the exercise is shown.

The cognitive exercise was based on the “go/no-go” paradigm, i.e., the participant has to respond when a “go” target appears; conversely, when a “no-go” target appears, participants should not react. During cycling, targets appear randomly at the edges of the path. The patient had to press the Bluetooth button mounted on the cycle-ergometer handlebar to kill the monsters (“go” targets), whereas animals (“no-go” targets) required no action (Figure 2). At each button press, the target disappeared, and visual feedback showed if the reaction was due or not. In case of a missed target, no feedback was provided.

The therapist could customize the ARTEDIA exercise by selecting, for each session, the level of difficulty from 1 to 3 (targets frequency and highlight), the cycle ergometer workload (30-70 W), and the duration of the exercise (10, 15, or 20 minutes). Also, the therapist could define the maximum HR (range: 70‐200 beats per minute) and minimum SpO_2_ values (range: 85%‐100%). The exercise was automatically interrupted if the physiological measurement overcame these safety thresholds for more than 10 seconds. At the end of each session, correct answers, errors, missed targets, average RPMs, HR, and SpO_2_ (if available) were saved.

### Participants

The participants in this study were patients in diverse conditions but suitable to receive an unsupervised training program following their Individual Rehabilitation Project (IRP). Enrolled patients were adults with post-COVID syndrome (INRCA) and stroke (VB), children, adolescents, and young adults with neuromotor diseases (MEDEA).

Participants had to be older than 12. Participants above legal age (18 years) had to be able to provide informed written consent; for children and adolescents below 18, the consent was obtained by their parent or legal guardian. All patients were selected among the ones referring to the 3 involved clinical centers and were judged in need of an additional training period after discharge. All the participants had to live in Lombardy in a house with an available space of 1.5×1 meters (to accommodate the cycle ergometer easily), own a good internet connection, and be assisted by a caregiver who could support home-based training. Exclusion criteria were pain; tracheostomy; reduced trunk control; history of seizure; severe respiratory, renal, hepatic, or cardiological conditions; and the presence of motor, sensory, or cognitive deficits preventing the execution of the exercises. Patients taking part in other rehabilitative programs were also excluded.

Sample size estimation was made based on the *attitude toward technology* questionnaire developed by Huygelier et al. [31] (see paragraph Outcomes). Considering an effect size of 0.69 (as in the original work, i.e., [31]), *α*=0.05, and power =0.9, we obtained a sample size of 24 patients.

### Study Protocol

#### Baseline

All patients were assessed for enrollment using the Trunk Control Test [32], the Visual Analog Scale for pain [33], and the Mini-Mental State Examination [34]. If considered eligible to participate in the study, each patient, depending on his or her condition, was administered a series of clinical measures to assess his or her baseline and define the IRP. These were the 6-minute walk test [35], the modified British Medical Research Council Questionnaire [36], the Quality of Upper Extremity Skills Test [37], the Gross Motor Function Measure [38], the Motricity Index [39], and the Berg Balance Scale [40].

Whenever possible, clinical scales were complemented with measures of cycling power to help the clinicians define ARTEDIA workload. Power measures were obtained using commercially available sensorized pedals (X-Power, SRM GmbH). This test saw 3 minutes of cycling on the cycle ergometer with a fixed workload set from 30 to 100 W. Data were analyzed to extract mean right and left power and the index of symmetry (*S*) [41]. The index of symmetry varies between 0 and 1, where 0 represents perfect symmetry, and 1 is the maximum power displacement (i.e., a leg has no or negative power).

#### Individual Rehabilitation Project

Depending on the baseline assessment, each patient was assigned to a complete telerehabilitation program, including lower limb aerobic and cognitive training with ARTEDIA, or an intervention foreseeing only Khymeia VRSS Home tablet exercises targeting cognitive and upper limb training.

The training was executed in unsupervised settings for all patients; most received the VRRS home tablet at their home. In total, 6 of them performed the rehabilitation program in a room that one of the clinics (VB) set up to execute the training program. Such a room was near the clinic but only opened, closed, and sanitized by non-medical personnel; thus, patients were completely autonomous in following their IRP.

Each patient had access to the telerehabilitation system from 15 to 60 days; patients were instructed to train as defined by the therapist in their IPR and set in their VRSS Home Tablet profile.

### Outcomes

Both subjective and objective outcomes were considered to achieve the objectives of the study.

The main outcome was attitude toward the technology; it was assessed before (T0) and after (T1) the intervention. This questionnaire, developed by Huygelier et al. [31], aimed at assessing the perceived ease of use, perceived usefulness, and anticipations about VR among older adults and was composed of 18 items to be assessed on a Likert scale from 1 (completely disagree) to 5 (completely agree).

Files stored on the VRSS home tablet were examined to extract objective data regarding patients’ adherence. From these files, we extracted the total number of sessions, the total exercise time per day, and the total time spent using ARTEDIA; the following parameters were available for each ARTEDIA exercise: duration, mean RPMs, mean HR, mean SpO_2_ (when present), number of correct answers, omissions, and errors.

As for subjective outcomes, the following dimensions were investigated at T1: perceived workload, usability, and technology acceptance. The questionnaires we used were the NASA Task Load Test (NASA-TLX) (considering raw scoring [42,43]), the System Usability Scale (SUS) [44], and the modified version of the Technology Acceptance Measure 3 (TAM3) [45] questionnaires, respectively.

NASA-TLX is a subjective method for measuring mental workload and includes 6 factors measured through a single question that has to be answered on a scale where 0 indicates no demand, and 20 maximum demand.

The TAM3 evaluates the acceptability of the system according to 13 dimensions. Responses to these questions were measured on a 5-point Likert scale, where 1 indicated strongly disagree and 5 strongly agree.

The SUS comprised 10 questions assessed on a 5-point Likert scale, ranging from 1 (strongly disagree) to 5 (strongly agree). The scores are totaled and then multiplied by 2.5 to calculate the final score, which ranges from 0 to 100. Higher scores reflect superior usability.

At the end of the experimental campaign, all the therapists involved in the study in the 3 clinical centers (n=7) were interviewed to provide feedback about the system. The questions of the semi-structured interview are reported in the Supplementary Materials in Multimedia Appendix 1.

### Statistical Analysis

Collected data are presented using medians and interquartile ranges; we report mean and standard deviations whenever needed for comparison with previous works. Pre- and post-intervention attitudes toward technology were compared using a paired Wilcoxon rank-sum test. Groups were compared using the Kruskal-Wallis test, and post hoc significance was investigated with Dunn’s test. Correlations among subjective variables and between subjective and objective variables were calculated using Spearman’s correlations. The reliability of TAM3 subscales was analyzed using Cronbach α. For all tests, the significance level was set at *P*<.05.

## Results

### Participant Characteristics

**Figure 3.**
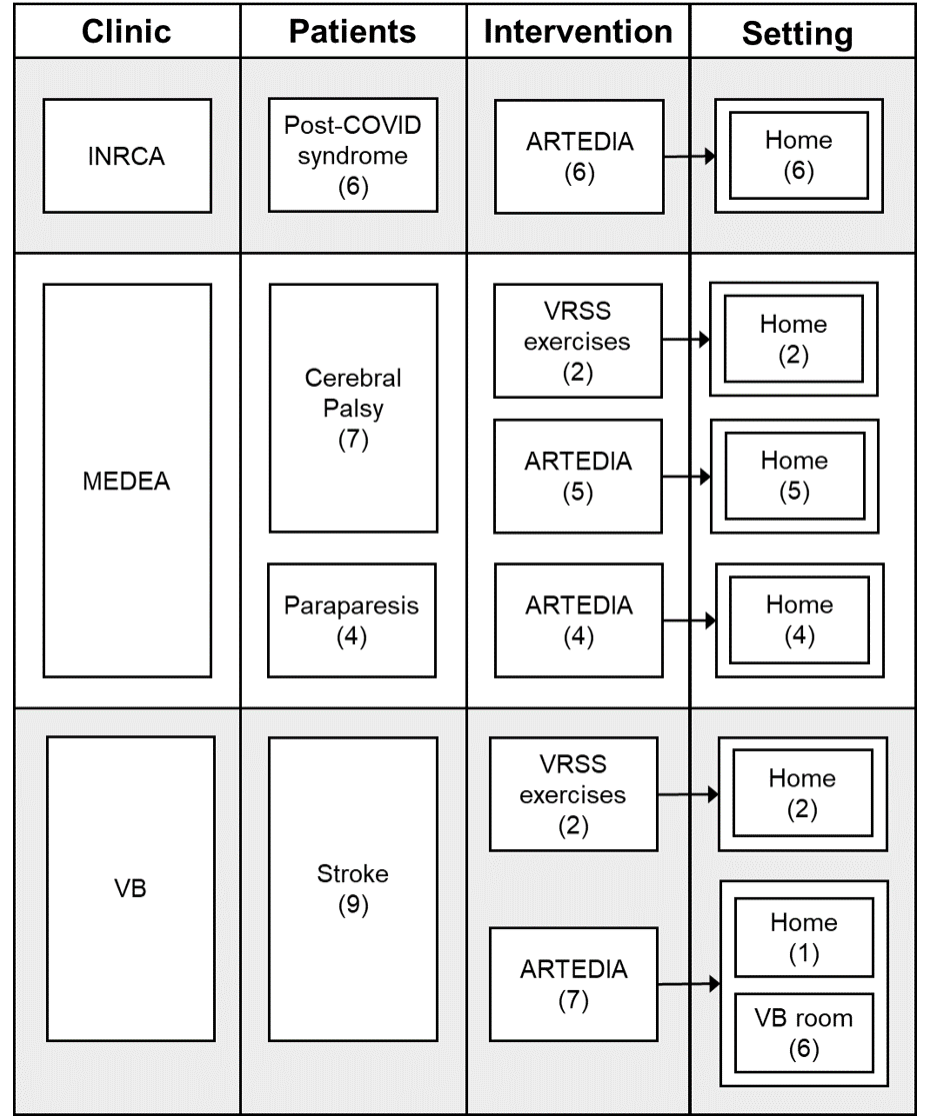
Final study allocation and the number of patients involved divided for any of the 3 clinics (INRCA, MEDEA, VB). In between brackets, the number of patients is reported. VRRS exercises indicate the exercises available on the Khymeia Home tablet; ARTEDIA stands for programs containing our newly developed exercise.

In total, 26 patients were enrolled in the 3 clinical centers. All participants had the maximum score in the Trunk Control Test and no pain, except for one (MEDEA2), who had a score of 2 due to foot pain. The patients’ final allocation and their health conditions are shown in Figure 3. Baseline data for each participant is reported in MultimediaAppendices 2^4^.

There were 3 dropouts. MEDEA8 (paraparesis) left the study because of the occurrence of pain, whereas VB008 was not able to cycle safely. One of the participants (VB006) had to leave the study after having performed 4 sessions of training due to a call for a surgical procedure, but questionnaire data were collected. All dropouts had the ARTEDIA exercise in their IRP.

### Attitude Toward Technology

**Figure 4.**
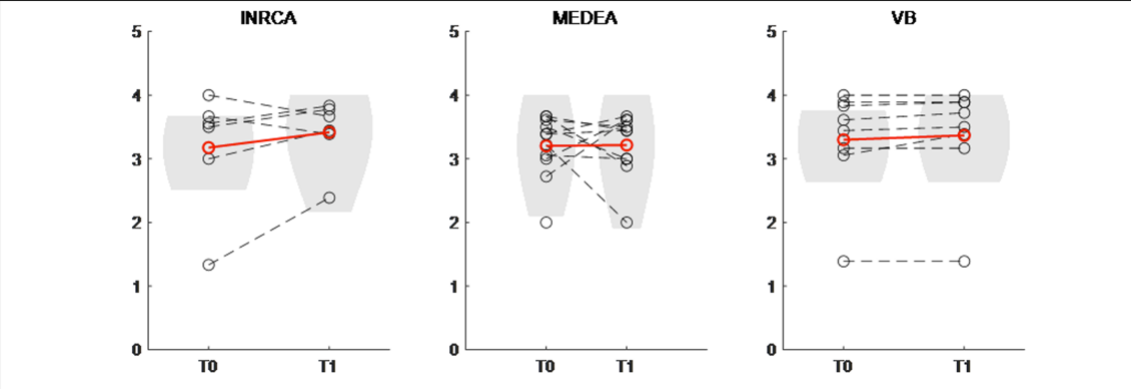
Attitude toward technology (range: 0‐5) in the 3 categories of patients as enrolled by the 3 clinics (INRCA, MEDEA, VB) measured pre- (**T0**) and post-intervention (**T1**). Each dashed gray line represents the observed scores of 1 participant, while the red solid line represents the group average. The gray area represents the density plots of the observed mean attitude scores.

The median attitude toward technology considering all patients was 3.44 (IQR 0.63, mean: 3.23 (SD 0.71)) out of 5 at T0 and 3.5 (IQR 0.48, mean: 3.40 (SD 0.48)) at T1. Figure 4 shows the attitude toward technology in the 3 categories of patients.

The attitude toward technology was positive (above 3) at T0 for most patients; such a value was preserved at T1 in most cases, especially among adults. One patient in the post-COVID group rated their attitude at T1 over 1 point with respect to T0 (INRCA2), and 1 patient with cerebral palsy reached 0.9 (MEDEA2). One young patient (MEDEA7) showed a decrease greater than 1 point at T1. No significant differences were recorded at the paired Wilcoxon test (*P*>.05), either considering the whole population of the patients enrolled by each clinic singularly.

### Objective Data

Table 1 contains data regarding the IRP of each patient and his or her hours of actual exercise with the Home Kit, considering both ARTEDIA and VRSS Home Tablet exercises. The adherence, calculated according to the IRP each patient received, was 0.59 (IQR 0.54, min: 0.16, max: 1).

**Table 1.** Information about each patient Individual Rehabilitation Project and their adherence to the overall program the therapist prescribed. Data regarding upper limb and cognitive training are presented for the Virtual Reality Rehabilitation System (VRSS); more details are provided for the ARTEDIA exercise.

				ARTEDIA			
ID patient	Hours ARTEDIA	Hours VRSS exercises	Adherence	Total number of sessions	No. days of training	No. sessions/ day	Mean duration/ day (min)
INRCA1	4.79	0	36%	15	14	1.14	21.71
INRCA2	6.54	0	49%	22	18	1.11	20.45
INRCA3	7.92	0	59%	24	18	1.28	75.24
INRCA4	3.77	0	100%	15	11	1.18	18.06
INRCA5	12.60	0	94%	38	36	1.03	20.44
INRCA6	12.85	0	28%	51	25	2.00	30.42
MEDEA1	8.19	11.45	98%	33	17	2.00	29.91
MEDEA2	6.44	11.97	92%	30	17	1.65	20.91
MEDEA3	0.00	12.95	65%	NA^a^	NA	NA	NA
MEDEA4	8.12	13.62	100%	31	16	1.88	29.98
MEDEA5	7.77	10.70	92%	32	17	1.76	26.69
MEDEA6	1.76	2.97	24%	6	4	1.00	18.52
MEDEA7	3.27	6.51	49%	17	7	2.14	26.36
MEDEA9	0.00	14.88	74%	NA	NA	NA	NA
MEDEA10	2.63	3.42	30%	11	10	1.00	15.04
MEDEA11	2.19	2.05	21%	10	5	1.80	25.08
VB001	0	19.67	89%	NA	NA	NA	NA
VB002	0	8.13	37%	NA	NA	NA	NA
VB003^b^	4.54	0	76%	18	9	1.78	26.86
VB004	5.48	0	52%	22	13	1.77	26.30
VB005^b^	0.70	0	16%	4	1	2.00	21.03
VB006^b^	2.87	0	72%	16	10	1.50	15.64
VB007^b^	4.49	0	41%	22	17.00	1.24	15.23
VB009^b^	^c^—	—	—	—	—	—	—

a NA: not applicable.

b patients who did the training in the clinic-devoted room.

c —: missing data.

No differences were present among groups considering total hours of actual training and adherence ratio. Nonetheless, the total hours planned by the therapists were different (H(2)= 8.57, *P*=.013), with patients from MEDEA receiving an IRP foreseeing significantly more hours of exercise than both INRCA (*P*=.04) and VB (*P*=.03). Table 2 displays data regarding the physical and cognitive performance of each patient who performed ARTEDIA (i.e., session with cycle ergometer) at least once.

**Table 2.** ARTEDIA performance considering all sessions. The correctness ratio was calculated by dividing the number of correct guesses by the total number of stimuli (targets or distractors).

	Physical performance						Cognitive performance	
ID patient	Mean WL^a^ (W)	Mean HR^b^ threshold (bpm)	Mean HR (bpm)	Mean SpO_2^c^_ threshold (%)	Mean SpO_2_ (%)	Mean RPM^d^	Level	Correctness ratio
INRCA1	40.67	128.67	51.84	90.00	97.60	63.18	2.87	0.96
INRCA2	42.73	130.32	87.77	90.32	88.87	64.21	2.91	0.91
INRCA3	30.83	130.00	113.73	90.00	94.41	78.01	3.00	0.98
INRCA4	70.00	132.94	98.17	NA^e^	NA	76.48	2.37	0.98
INRCA5	33.68	131.32	80.58	90.00	93.95	65.05	2.26	0.98
INRCA6	30.00	120.00	97.84	90.00	74.80	62.37	3.00	0.91
MEDEA1	54.85	176.97	150.72	NA	NA	68.63	3.00	0.99
MEDEA2	62.33	180.00	123.59	NA	NA	64.47	1.00	0.95
MEDEA4	30.32	180.00	136.49	NA	NA	63.66	2.94	0.95
MEDEA5	59.69	180.00	113.19	NA	NA	60.91	2.66	0.98
MEDEA6	30.00	180.00	115.90	NA	NA	39.69	1.00	0.80
MEDEA7	52.35	180.00	101.25	NA	NA	63.47	1.12	0.92
MEDEA10	30.00	180.00	133.05	NA	NA	61.03	1.91	0.48
MEDEA11	52.00	180.40	112.10	NA	NA	63.41	1.80	0.65
VB003	30.00	120.00	99.76	NA	NA	62.96	1.00	0.99
VB004	30.00	137.27	81.44	NA	NA	48.46	1.00	0.95
VB005	30.00	125.00	112.99	NA	NA	62.16	1.00	1.00
VB006	30.00	132.19	74.43	NA	NA	65.14	1.00	0.94
VB007	30.00	132.73	88.21	NA	NA	75.25	1.00	0.98
VB009	—	—	—	NA	NA	—	—	—

a WL: workload.

b HR: heart rate.

c SpO_2_: oxygen saturation.

d RPM: rounds per minute.

e NA: not applicable.

### Subjective Data

**Figure 5.**
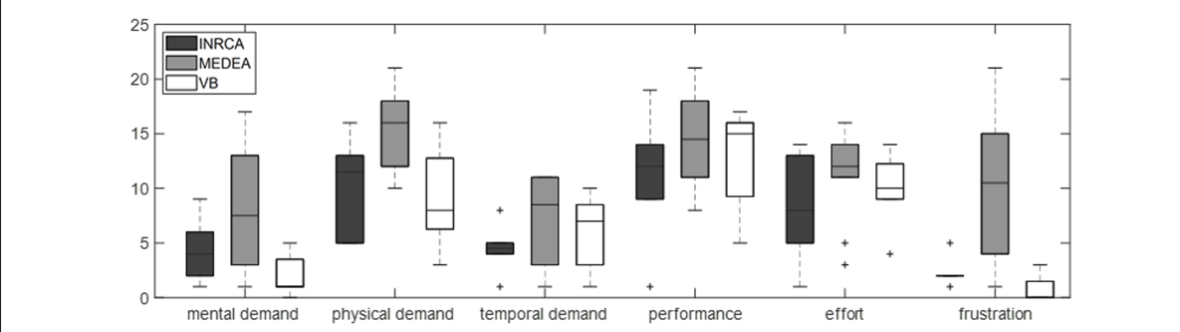
Perceived workload in the 3 populations of patients as enrolled by the 3 clinics (INRCA, MEDEA, VB). The reported items are the subscales measured by the NASA-Task Load IndeX (TLX).

**Figure 6.**
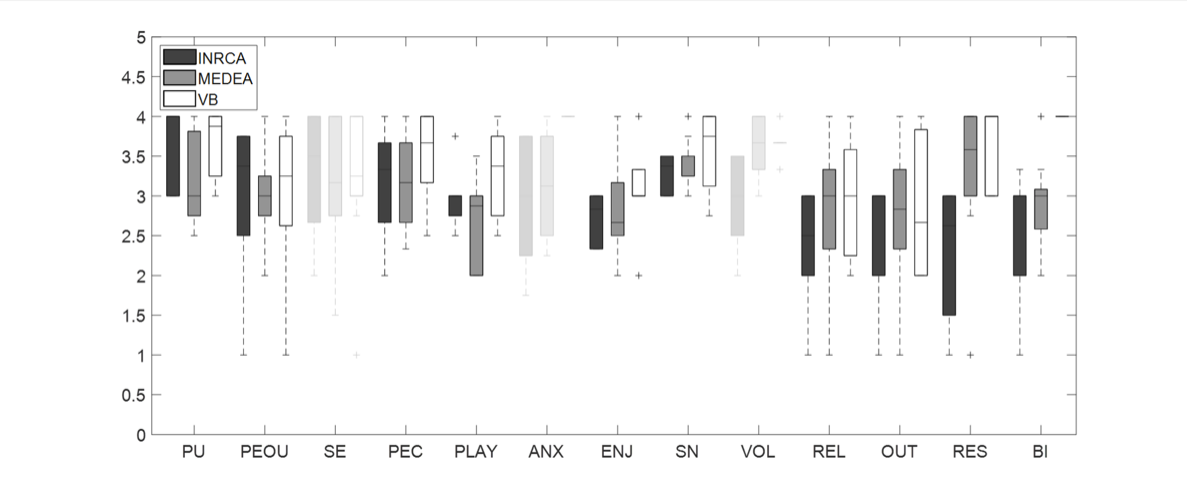
TAM3 subscale values in the 3 populations of patients as enrolled by the 3 clinics (INRCA, MEDEA, VB). Non-reliable scales are grayed out. PU: perceived usefulness; PEOU: perceived ease of use; SE: self-efficacy; PEC: perception of external control; PLAY: playfulness; ANX: anxiety; ENJ: enjoyment; SN: subjective norm; VOL: voluntariness; REL: relevance; OUT: output quality; RES: result demonstrability; BI: behavioral intention.

The workload measured at T1 is shown in Figure 5. Usability score was found to be in the “acceptable” and “excellent” ranges according to [46]; its median value was 80 (IQR 20, mean 75.73 (SD 17.45)). Figure 6 and Table 3 depict the correlations among TAM3 subscales and their reliability.

**Table 3.** Correlations among TAM3 subscales and their reliability.

Variable	PU^a^	PEOU^b^	SE^c^	PEC^d^	PLAY^e^	ANX^f^	ENJ^g^	SN^h^	VOL^i^	REL^j^	OUT^k^	RES^l^	BI^m^	α
PU														
r_s_		0.41	0.33	0.34	0.35	0.16	0.35	0.07	0.23	0.36	0.31	0.08	0.25	0.97
*P* value		.07	.15	.14	.13	.51	.13	.78	.32	.12	.18	.72	.30	
PEOU														
r_s_			0.72	0.62	0.19	0.60	0.31	-0.24	0.34	0.22	0.36	0.51	0.47	0.87
*P* value			<.001	<.001	.42	<.001	.19	.31	.14	.34	.12	.02	.04	
SE														
r_s_				0.69	0.28	0.48	0.29	-0.04	0.36	0.38	0.42	0.42	0.49	0.56
*P* value				<.001	.24	.03	.21	.85	.12	.10	.06	.07	.03	
PEC														
r_s_				-	0.35	0.51	0.38	0.11	0.58	0.28	0.53	0.63	0.34	0.62
*P* value					.13	.02	.09	.65	.01	.24	.02	<.001	.14	
PLAY														
r_s_						0.13	0.64	0.28	0.18	0.59	0.62	0.43	0.37	0.70
*P* value						.60	<.001	.24	.45	.01	<.001	.06	.11	
ANX														
r_s_							0.45	0.18	0.67	0.31	0.22	0.64	0.75	0.51
*P* value							.05	.46	<.001	.18	.34	<.001	<.001	
ENJ														
r_s_								0.10	0.49	0.65	0.70	0.50	0.50	0.86
*P* value								.67	.03	<.001	<.001	.02	.03	
SN														
r_s_								-	0.25	0.34	0.06	0.18	0.54	0.64
*P* value									.29	.15	.81	.44	.01	
VOL														
r_s_									-	0.32	0.43	0.62	0.45	0.25
*P* value										.17	.06	<.001	.05	
REL														
r_s_										-	0.78	0.50	0.62	0.87
*P* value											<.001	.03	<.001	
OUT														
r_s_											-	0.59	0.37	0.91
*P* value												.01	.11	
RES														
r_s_												-	0.59	0.91
*P* value													.01	
BI														
r_s_													-	0.95
*P* value														

a PU: perceived usefulness.

b PEOU: perceived ease of use.

c SE: self-efficacy.

d PEC: perception of external control.

e PLAY: playfulness.

f ANX: anxiety.

g ENJ: enjoyment.

h SN: subjective norm.

i VOL: voluntariness.

j REL: relevance.

k OUT: output quality.

l RES: result demonstrability.

m BI: behavioral intention.

In terms of subjective outcomes, group differences emerged in terms of perceived cognitive workload, in particular in the subscales of physical demand (H(2)=8.00, *P*=.018) and frustration (H(2)=11.36, *P*=.035); MEDEA reached higher levels than VB for both variables (*P*=0.03and *P*=.002, respectively). Significant differences among groups were also present in the case of technology acceptance, in particular for the subscales of result demonstrability (H(2)=13.13, *P*=.001) and behavioral intention (H(2)=11.76, *P*=.003). Post-hoc tests showed that VB results were more positive than both MEDEA and INRCA (*P*<.007 in all cases). No other significant group differences were present.

Mean attitude toward technology at T1 was negatively correlated with physical demand (r_s_=−0.48, *P*=.018), effort (r_s_=−0.41, *P*=.049), and frustration (r_s_=−0.52, *P*=.01) as measured by the NASA-TLX. Mean attitude at T0 was significantly correlated with frustration only (r_s_=−0.51, *P*=.01).

Mean attitude (T1) and technology acceptance subscale correlations were significant (*P*<.05) in the case of perceived usefulness (r_s_=0.52), perceived ease of use (r_s_=0.55), playfulness (r_s_=0.50), relevance (r_s_=0.44), and behavioral intention (r_s_=0.50); the correlation of attitude with usability was significant (r_s_=0.68, *P*<.001).

None of the subjective variables was correlated to total hours of training or adherence ratio.

Considering those who did ARTEDIA, the difference between the HR threshold and the mean HR measured throughout the sessions was correlated only with frustration (r_s_=0.55, *P*=.017). The correctness ratio (i.e., the number of targets identified correctly divided by the total targets) correlated significantly and negatively with mental demand and frustration (r_s_=−0.62, *P*=.007 and r_s_=−0.53, *P*=.023, respectively). No other correlation was significant.

### Therapists’ Feedback

The results of the semi-structured interview are presented in Table 4.

**Table 4.** The therapists’ feedback grouped according to themes and positive and negative comments. When the comments were related to one participant in particular, their ID is reported between brackets.

	Pros	Cons
Perceived usefulness	Implementation of continuity of care Home-based treatment favored compliance Possibility of lower limb training Patients’ monitoring throughout time	Exercises are generic and not well-targeted No easy assessment of the performance quality (i.e., if compensation was used) Impossible to establish the reason behind pain occurrence (MEDEA7)
Accessibility	Accessibility for chronic patients not receiving other care Accessibility for patients living in remote areas	Patients must fit precise conditions (high cognitive functioning, no spasticity) Patients must be available to schedule calls with the therapist during working hours (MEDEA11) The safety thresholds were not suitable for one patient (INRCA4) Internet connection was not always stable enough Architectural barriers impeded the installation of the setup (stairs, lack of space)
Usability	Good autonomous management in most cases Scheduling daily exercises promoted adherence Engaging scenario promoting motivation	Technical issues Difficulty in providing support in case of malfunctioning

As per technical issues, we have tracked the following occurrences in patients from MEDEA: the sensors were not responding properly or were sending wrong input data (n=1; MEDEA7); server updates did not allow the therapist to update the IRP and the patient to access the daily training (n=2; MEDEA4, MEDEA5); generic malfunctioning of the system (n=1, MEDEA6). For one post-COVID patient (INRCA4), the minimum threshold set for oxygen saturation was still too high to proceed with the training. In this case, the patient replaced the sensor with another that was not linked to the system and monitored themselves according to the therapist’s indications. Insufficient power of the internet connection and architectural barriers were mentioned in all the interviews.

## Discussion

### Principal Findings

The results of this study indicated the feasibility and acceptability of a customized VR-based telerehabilitation program that allowed for the safe implementation of lower limb and aerobic training. This result aligns with the outcomes in previous works studying telerehabilitation interventions, either with or without VR support. Furthermore, conducting this study allowed the provision of rehabilitation services to patients who had limited access to the clinic due to geographical distance or had ended their hospitalization.

The study was performed by enrolling a heterogeneous sample of patients, comprising post-COVID patients and adults, adolescents, and children with neuromotor impairments. It allowed us to demonstrate the feasibility of our proposed customizable telerehabilitation program to address diverse issues and needs. Although heterogeneity may constitute a limitation, it can be welcomed when scientific (e.g., pragmatic) studies are aimed to demonstrate the effectiveness in a population as similar as possible to the target population who may benefit from the intervention [47]. Moreover, it could help design the future delivery of targeted care, accounting for patients’ diverse conditions [48].

Regarding the attitude toward technology, contrary to what was found in [31], in which Huygelier et al. recorded an increase from 3.4 to 3.9, we did not find any improvement after the treatment. This outcome may be explained by considering that the original work used immersive VR, whereas our system used more familiar technologies, such as laptops and smartphones. Therefore, it is possible that our participants did not change their minds due to the lack of a “wow” effect [49]; also, their anticipation and perception toward the use of digital technology were probably correct at the baseline and confirmed at the end of the experience [50]. Nonetheless, the patients’ relationship and approach to technology did not change, and no technical factors affected technology adoption and caused participants to leave the program. This was also confirmed by usability and acceptance outcomes, which were both satisfactory (all technology acceptance subscale scores were >2.7, up to 5), and the positive correlations, found at T1, between attitude and usability, and attitude and perceived ease of use, playfulness, and intentions to reuse the system.

On the other hand, as mentioned by the therapists in their interview, our sample was selected to fit the requirements of mostly autonomous system use (i.e., cognitive level and presence of a caregiver), thus introducing a possible bias in this sense. Indeed, literature shows that the general acceptance of mHealth applications still encounters patients’ resistance in more than half of the cases, resulting in minimal engagement and no clinical improvements [51,52].

Our patients’ adherence to the proposed program was moderate-to-good (59%), in line with other works proposing unsupervised physical activity programs [53,54] or rehabilitation activities for patients [55]. Other interventions indeed recorded higher adherence, but they also accounted for a stricter sample selection: e.g., a tablet-guided exercise program for older adults obtained 85% adherence, but only 36% of patients accepted to participate in the program and completed it [56].

This issue has already been discussed in the literature, especially when dealing with programs promoting physical exercise: participants in scientific studies are volunteers and, therefore, inherently motivated to exercise or willing to be involved. This means that the results of trials engaging patients in exercise are mostly not generalizable to the general population.

Previous work tried to identify the factors that may influence adherence; a review conducted in 2021 identified 14 key factors affecting adherence in rehabilitation programs [57]; among the others, there was the involvement of professionals from several disciplines, exploration of patients’ barriers and facilitators, participants’ education, integration in daily living and social support, which we did not investigate. Among older adults, factors promoting an active lifestyle were also identified as personal factors: younger age, education, history of regular exercise, less depression, fewer impairments, and chronic conditions could all positively influence adherence to training programs [58,59].

This dependence upon personal factors was possibly present also in our case, as individual differences, not detected by our collected data, were present. Only in a few cases, analyzing all the information together, was it possible to formulate a hypothesis for higher or lower adherence. For instance, MEDEA7 encountered some problems with the sensors and experienced footache, and the therapist was not able to investigate the cause of the pain remotely. This has impacted their attitude toward technology (showing a decrease of over 1 point from T0 to T1) and possibly their adherence (49%). In the case of INRCA6, MEDEA6, MEDEA 10, and VB002, it is plausible that their initial condition—worse than other peers’—negatively impacted adherence, as they had to make more effort to participate in the program.

These considerations are to be considered cautiously: they are hypotheses, and in most cases, both the adherence and the total training hours were not directly attributable to any of the variables investigated. Because of this, we encourage future work to focus more on personal and anamnestic characteristics we have not explored.

Another element that could contribute to assessing the acceptability of an intervention and influencing patients’ adherence is workload, i.e., the cost incurred by an individual while achieving a certain performance on a task with specific demands [60]. The concept of workload per se is hard to interpret, as it is impossible for many tasks to define a “redline” above which the performance degrades [61].

Indeed, in our case, it is plausible to think that some mental effort would be required to perform the proposed exercises per se, even without any influence of the technological system. Considering the performance data related to ARTEDIA dual-task exercise, it emerged that lower cognitive results were indeed linked to higher cognitive demands.

Instead, both cognitive and physical performances were negatively correlated with frustration. In the NASA-TLX questionnaire, the frustration item foresees the investigation of “how insecure, discouraged, irritated, and annoyed” one feels while accomplishing a task, i.e., it examines the unsuccessful fulfillment of objectives after repetitive attempts due to obstacles and negative feedback [62]. Also in this case, it is impossible to discriminate precisely if the frustration was due to the task itself (either cycling or go/no-go task) or the system management. However, some considerations could be made by examining the significant differences among groups: patients from MEDEA reported more frustration and physical effort and were also the group reporting the higher occurrence of problems, such as VRRS updates that caused temporary unavailability of the system, sensor malfunctioning, and the impossibility of the therapist to assess the cause of pain remotely. The relationship between frustration and technical issues has emerged before [63,64].

Another consideration that could be made is related to the absolute dose of therapy: the group of younger participants from MEDEA was also the one receiving a more intense training plan. Although no correlations were identified (possibly because of the small sample), this outcome was in agreement with previous studies, which highlighted that lower-dose interventions could fit better the patients’ schedules and, therefore, be more accepted [65,66]. This was highlighted by the case of MEDEA11, who was mostly unavailable for the video calls with the therapists and had a low adherence rate, possibly because of other duties of daily living (21%). Given this, the topic of the appropriate dose to promote adherence in telerehabilitation deserves to be further explored in future works.

On the contrary, VB patients were less subjected to problems, possibly because the system was regularly checked and sanitized (as it was shared and close to the clinic, with one exception only), and—as mentioned before—they trained significantly less.

Despite the reduced adherence, due possibly to the need to travel to reach the room close to the clinic, the hybrid solution has been appreciated and, therefore, deserves further analysis, especially in the case of non-intensive training, e.g, for maintenance of muscle fitness and promotion of an active lifestyle among chronic patients.

In such a setting, sharing a single piece of equipment saves personnel time, money, and equipment management duties (e.g., handling transfers, installations, and conformity checks at the end of the training). Plus, environmental barriers such as lack of space and adequate internet connection are excluded, and prompt support can be provided in the case of technical or clinical issues. On the other hand, the accessibility to the service remains available only for those patients who can travel and reach the facility.

### Limitations

This work has some limitations. First of all, the sample was heterogeneous, and although this choice allowed testing our telerehabilitation program with diverse conditions, this limited the generalization to the populations of adults and children with neuromuscular or post-COVID-related impairments. Second, the adherence to the intervention was very different, and we could not determine which factors influenced it either positively or negatively. Finally, rehabilitation sessions were carried out in two different scenarios (home and clinic-devoted room), and study participants were part of different age groups; this may have influenced the patients’ subjective experiences. Although the in-depth evaluation of differences among subgroups goes beyond the scope of this work, it would have been interesting to investigate the effects of other factors, such as age or setting. Unfortunately, this was not possible due to the small sample. Nonetheless, the preliminary assessment of the feasibility of the proposed intervention paves the way for future trials in which more participants per group should be enrolled.

### Conclusions

This work presents a feasibility study of a novel telerehabilitation system allowing cycling-based dual-task training. The system was preliminarily assessed with a sample of patients with different conditions and demonstrated usable, meaningful, and acceptable. Moderate acceptance was found by therapists who recognized several strengths but also a number of issues. For the effective implementation of a complete telerehabilitation program, including lower limbs, some improvements are still required to overcome environmental and technical barriers.

In the future, larger and more homogeneous populations should be engaged to extend the obtained results and increase their generalizability. A higher number of participants will also allow us to delve deeper into subgroup differences, highlighting which features are considered more relevant by each patient or age group to enhance personalization even more. Particular attention should be paid to the therapy dose and the rehabilitation setting to find the correct balance between treatment effectiveness and suitability with patients’ daily life in order to promote optimal compliance.

## Supplementary material

10.2196/71099 Multimedia Appendix 1 Questionnaire for the therapists.

10.2196/71099 Multimedia Appendix 2 Characteristics of INRCA participants at the baseline; 6MWT: 6 minutes walking test in meters and velocity; mMRC: modified British Medical Research Council Questionnaire; S: index of symmetry; Pr/Pl: mean cycling power of right/left leg.

10.2196/71099 Multimedia Appendix 3 Characteristics of MEDEA participants at the baseline; *: drop-out; 6MWT: 6 minutes walking test in meters; QUEST: Quality of Upper Extremity Skills Test, only administered in participants with upper limb impairments; GMFM: Gross Motor Function Measure, administered only for children below 18 years old; S: index of symmetry; Pr/Pl: mean cycling power of right/left leg. NA: Not Applicable; /: missing value.

10.2196/71099 Multimedia Appendix 4 Characteristics of VB participants at the baseline; *: drop out; S: index of symmetry; Pr/Pl: mean cycling power of right/left leg. NA: Not Applicable; /: missing value.

## Abbreviations

ANX: anxiety
BI: behavioral intention
ENJ: enjoyment
HR: heart rate
INRCA: IRCCS INRCA Casatenovo
IRP: Individual Rehabilitation Project
MEDEA: IRCCS E. Medea
NASA-TLX: Nasa Task Load Index
OUT: output quality
PEC: perception of external control
PEOU: perceived ease of use
PLAY: playfulness
PU: perceived usefulness
REL: relevance
RES: result demonstrability
RPM: revolutions per minute
SE: self-efficacy
SN: subjective norm
SUS: System Usability Scale
TAM3: Technology Acceptance Measure 3
VB: Villa Beretta Rehabilitation Center
VOL: voluntariness
VR: virtual reality
VRRS: Virtual Reality Rehabilitation System
