# Supplementary material for: Cycling-Based Telerehabilitation: Acceptability and Feasibility Study
Source: JMIR Hum Factors. 2025 Sep 10;12:e71099. doi: 10.2196/71099 (PMC12422529; doi:10.2196/71099)
Supplement: Multimedia Appendix 1 [file humanfactors-v12-e71099-s001.docx]

**Supplementary material**

**Perceived usefulness**

1. In your opinion, is this telerehabilitation program useful for the patient? Why?
2. Which aspects of this program do you consider meaningful (e.g., motivation, constant monitoring, home-based training)?
3. Do you think using this telerehabilitation platform would ease your job? How?

**Accessibility and barriers**

1. What are the advantages that the use of this platform can bring to patients?
2. Do you think using the telerehabilitation platform complicate some aspects of your job or some rehabilitation processes?
3. Are there barriers limiting the widespread of this telerehabilitation platform?

**Usability**

1. Is this system easy to use?
2. Are there aspects that have been hard to learn? Which ones?
3. Did you have to learn many tasks to operate the platform autonomously?
