# Supplementary material for: Cycling-Based Telerehabilitation: Acceptability and Feasibility Study
Source: JMIR Hum Factors. 2025 Sep 10;12:e71099. doi: 10.2196/71099 (PMC12422529; doi:10.2196/71099)
Supplement: Multimedia Appendix 2 [file humanfactors-v12-e71099-s002.docx]

Supplementary Table 1. Characteristics of INRCA participants at the baseline; 6MWT: 6 minutes walking test in meters and velocity; mMRC: modified British Medical Research Council Questionnaire; S: index of symmetry; Pr/Pl: mean cycling power of right/left leg.

| **ID** | **age** | **gender** | **Clinical condition** | **Months since COVID** | **6MWT**  **(m [k/h])** | **mMRC** | **S** | **P_r_/P_l_ (W)** |
| --- | --- | --- | --- | --- | --- | --- | --- | --- |
| INRCA1 | 55 | M | Post-COVID | 33 | 616 (6.8) | 1 | 0.06 | 38/43 |
| INRCA2 | 54 | M | Post-COVID | 27 | 532 (5.9) | 2 | 0.28 | 25/44 |
| INRCA3 | 72 | M | Post-COVID | 27 | 608 (5.6) | 1 | \ | \ |
| INRCA4 | 63 | M | Post-COVID | 24 | 398 (5.04) | 2 | \ | \ |
| INRCA5 | 56 | M | Post-COVID | 36 | 524 (5.6) | 2 | \ | \ |
| INRCA6 | 75 | F | Post-COVID | 36 | 670 (9.1) | 2 | \ | \ |
