# Supplementary material for: Cycling-Based Telerehabilitation: Acceptability and Feasibility Study
Source: JMIR Hum Factors. 2025 Sep 10;12:e71099. doi: 10.2196/71099 (PMC12422529; doi:10.2196/71099)
Supplement: Multimedia Appendix 3 [file humanfactors-v12-e71099-s003.docx]

Supplementary Table 2. Characteristics of MEDEA participants at the baseline; *: drop-out; 6MWT: 6 minutes walking test in meters; QUEST: Quality of Upper Extremity Skills Test, only administered in participants with upper limb impairments; GMFM: Gross Motor Function Measure, administered only for children below 18 years old; S: index of symmetry; Pr/Pl: mean cycling power of right/left leg. NA: Not Applicable; /: missing value.

| **ID** | **age** | **gender** | **Clinical condition** | **side** | **6MWT (m)** | **GMFM (%tot)** | **QUEST (%tot)** | **S** | **Pr/Pl (W)** |
| --- | --- | --- | --- | --- | --- | --- | --- | --- | --- |
| MEDEA1 | 15 | F | CP | Left | 566.4 | 99 | NA | 0.47 | 45/16 |
| MEDEA2 | 13 | M | CP | Right | 572.7 | 99 | 78.34 | 0.36 | 22/48 |
| MEDEA3 | 19 | M | CP | Bilateral | 229 | NA | NA | / | / |
| MEDEA4 | 20 | F | CP | Bilateral | 414 | NA | 87.7 | / | / |
| MEDEA5 | 16 | M | CP | Right | 417 | 93 | 78.75 | 0.11 | 35/43 |
| MEDEA6 | 23 | M | Paraparesis | Bilateral | 292.4 | NA | NA | 0.29 | 21/12 |
| MEDEA7 | 17 | F | CP | Bilateral | 432.3 | 81 | 87.83 | 0.24 | 25/40 |
| MEDEA8* | 18 | M | Paraparesis | Bilateral | 325.5 | / | / | / | / |
| MEDEA9 | 28 | M | CP | Bilateral | 425 | NA | NA | / | / |
| MEDEA10 | 18 | F | Paraparesis | Bilateral | 323.6 | NA | NA | 0.05 | 29/24 |
| MEDEA11 | 25 | M | Paraparesis | Bilateral | 507.5 | NA | NA | 0.001 | 37/38 |
