# Supplementary material for: Cycling-Based Telerehabilitation: Acceptability and Feasibility Study
Source: JMIR Hum Factors. 2025 Sep 10;12:e71099. doi: 10.2196/71099 (PMC12422529; doi:10.2196/71099)
Supplement: Multimedia Appendix 4 [file humanfactors-v12-e71099-s004.docx]

Supplementary Table 3. Characteristics of VB participants at the baseline; *: drop out; S: index of symmetry; Pr/Pl: mean cycling power of right/left leg.

| **ID** | **age** | **gender** | **Clinical condition** | **Stroke occurrence** | **Motricity index** | **Berg Balance**  **Scale** | **S** | **Pr/Pl (W)** |
| --- | --- | --- | --- | --- | --- | --- | --- | --- |
| VB001 | 48 | M | stroke | 09/02/2022 | 71 | 42 | **/** | **/** |
| VB002 | 58 | F | stroke | 09/04/2017 | 76 | 16 | **/** | **/** |
| VB003 | 48 | M | stroke | 09/02/2022 | 76 | 43 | 1 | -28/90 |
| VB004 | 58 | F | stroke | 09/04/2018 | 76 | 26 | **/** | **/** |
| VB005 | 38 | M | stroke | 15/02/2022 | 65 | 53 | **/** | **/** |
| VB006 | 79 | M | stroke | 07/12/2022 | 76 | 52 | **/** | **/** |
| VB007 | 41 | M | stroke | 25/09/2022 | 54 | 46 | **/** | **/** |
| VB008* | 76 | F | stroke | 29/07/2021 | 58 | 40 | **/** | **/** |
| VB009 | 71 | M | stroke | 25/06/1905 | 29 | 16 | **/** | **/** |
